# *H influenzae* LPS colocalization with Toll-like receptor 4 in eosinophilic esophagitis

**DOI:** 10.1016/j.jacig.2023.100151

**Published:** 2023-07-20

**Authors:** Anupama Ravi, Eric V. Marietta, Jeffrey A. Alexander, Joseph A. Murray, David A. Katzka

**Affiliations:** a Division of Pediatric Allergy, Mayo Clinic, Rochester; b Division of Gastroenterology and Hepatology, Mayo Clinic, Rochester; c Division of Digestive and Liver Disease, Columbia University, New York

**Keywords:** Eosinophilic esophagitis, microbiome, LPS, Toll-like receptor, esophagus, esophagitis

## Abstract

**Background:**

Patients with eosinophilic esophagitis (EoE) have a unique esophageal microbiome with increased presence of *Haemophilus influenzae*, but its role in the disease is unclear.

**Objective:**

Microbiome-derived bacterial LPS activation of Toll-like receptors (TLR) is a potential mechanism for inducing inflammation in other chronic inflammatory diseases, but it has not been studied in EoE. Our aim was therefore to study microbiome-derived bacterial LPS activation of TLRs in EoE.

**Methods:**

We studied 10 patients with active EoE, 9 patients with inactive EoE, and 10 control patients. Esophageal biopsy samples from the controls, patients with active EoE (>15 eosinophils/hpf), and patients with inactive EoE were immunostained for the presence of *H influenzae* LPS, presence of TLR4, and colocalization of LPS and TLR4. Staining intensity was measured by using confocal laser microscopy and scored on a scale from 0 to 3 as the average score assigned by 2 blinded observers.

**Results:**

*H influenzae* LPS was detected by positive staining in 20 of the 29 patients (69.0%), including 9 of the 10 patients with active EoE (90.0%), 8 of the 9 patients with inactive EoE (89.9%), and 3 of the 10 controls (30%); its level was greater in the patients with active EoE than in the controls (*P* = .063). TLR4 was detected by positive staining in 19 of the 29 patients (65.5%), including 9 of the 10 patients with active EoE (90.0%), 4 of the 9 patients with inactive EoE (44.4%), and 6 of the 10 controls (60.0%); its level was higher in the patients with active EoE than in those with inactive EoE (*P* = .096). The result of testing for colocalization of LPS and TLR4 was positive in 8 of 10 patients with active EoE (80.0%), 1 of 9 patients with inactive EoE (11.1%), and 1 of 10 control patients (10.0%), with greater colocalization of *H influenzae* LPS and TLR4 staining density in the samples from patients with active EoE than in the controls or the patients with inactive EoE (*P* = .009 and *P* = .018, respectively).

**Conclusion:**

Esophageal microbiome–rich *H influenzae* LPS colocalizes to TLR4 in active EoE. These data lend further support to a role for the esophageal microbiome in modulating the activity of EoE.

## Introduction

Eosinophilic esophagitis (EoE) is an immune-mediated inflammatory disease, characterized by symptoms of esophageal dysfunction and eosinophil-predominant inflammation. There are several contributing etiologies to eosinophilic esophagitis, including genomic and allergic factors.^1^ An evolving area in EoE pathogenesis is the microbiome. Several studies have demonstrated abnormalities in both the esophageal and gut microbiome.^2^, ^3^, ^4^, ^5^ For example, 1 study demonstrated a marked decrease in Firmicutes and an increase in Bacteroidetes, and at the order and family levels, there were significant decreases in Clostridia and Clostridiales in the stool.^6^ In a study of patients with EoE who were and were not currently undergoing treatment, differential testing of microbial relative abundance displayed significant changes for the genera *Filifactor, Parvimonas,* and *Porphyromonas*.^7^ The mechanism by which an abnormal microbiome predisposes to immune dysfunction in EoE could include bacterial breakdown of food antigens and/or permeation of bacterial-derived antigenic proteins. One such protein is LPS.

LPSs are endotoxins produced by and present on the surface of bacteria and capable of activating the immune system.^8^ Specific bacteria of the microbiome may produce endogenous LPS. One of these bacteria is *Haemophilus influenzae*, a bacterium endemic to the esophageal microbiome in normal^9^ and inflammatory conditions.^2^ Additionally, an increased abundance of *H influenzae* in the esophagus has been demonstrated in patients with active EoE.^5^ *H influenzae* in the airway microbiome is also thought to be a mediator of TLR activity in asthma.^10^ The release of LPS into the mucosa may activate proinflammatory lymphocytes and TLR4.^11^ TLRs activate antigen-presenting cells and regulatory T cells, which are critical in the immune pathway. They are also associated with other allergic diseases such as atopic dermatitis.^12^ In EoE, active disease is associated with increased TLRs, including TLR4.^13^ The aim of this study was to assess the physical association of TLR4 with LPS in patients with active and inactive eosinophilic esophagitis.

## Results and discussion

For a detailed description of the immunostaining protocol, see the Supplementary Methods in the Online Repository at www.jaci-global.org.

**Fig 1:**
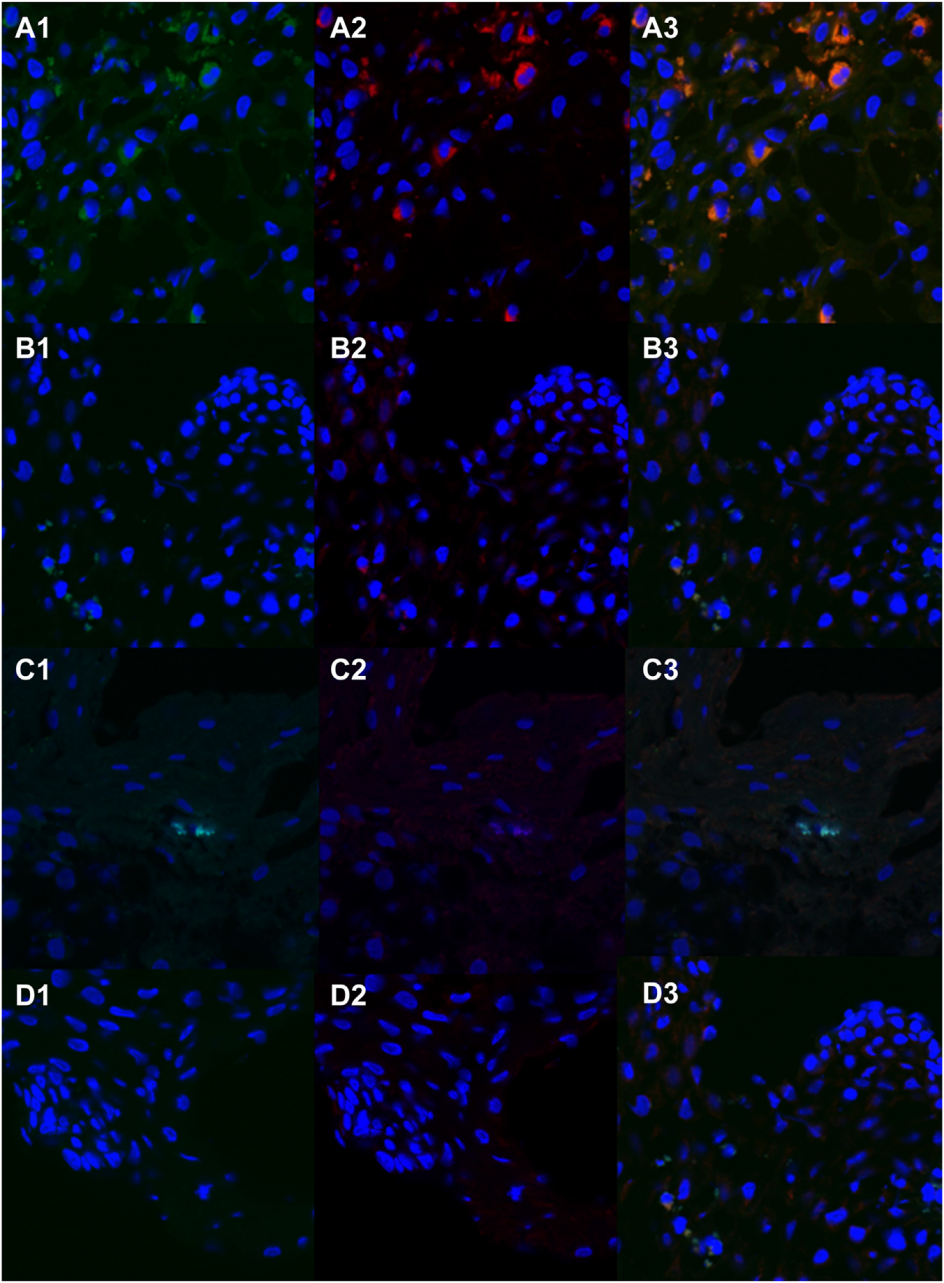
Immunostaining of *H influenzae* LPS and TLR4 in the esophageal mucosa of the patients with EoE and the control patients. **A,** H influenzae LPS (score 3) (**A1**), TLR4 (score 3) (**A2**), and Colocalization of H influenzae LPS and TLR4 (score 3) (**A3**) in a patient with active EoE. **B,***H influenzae* LPS (score 1) (**B1**), TLR4 (score 2) (**B2**), and colocalization of *H influenzae* LPS and TLR4 (score 2) in a patient with active EoE (**B3**). **C,***H influenzae* LPS (score 1) (**C1**), TLR4 (score 0) (**C2**), and colocalization of *H influenzae* LPS and TLR4 (score 0) in a patient with inactive EoE (**C3**). **D,***H influenzae* LPS (score 0) (**D1**), TLR4 (score 0) (**D2**), and colocalization of *H influenzae* LPS and TLR4 (score 0) in control patient (**D3**). *H influenzae* LPS (*green*), TLR4 (*red*), colocalization of LPS and TLR4 (*orange*), and nucleic acid (*blue*). Original magnification, ×40.

**Fig 2:**
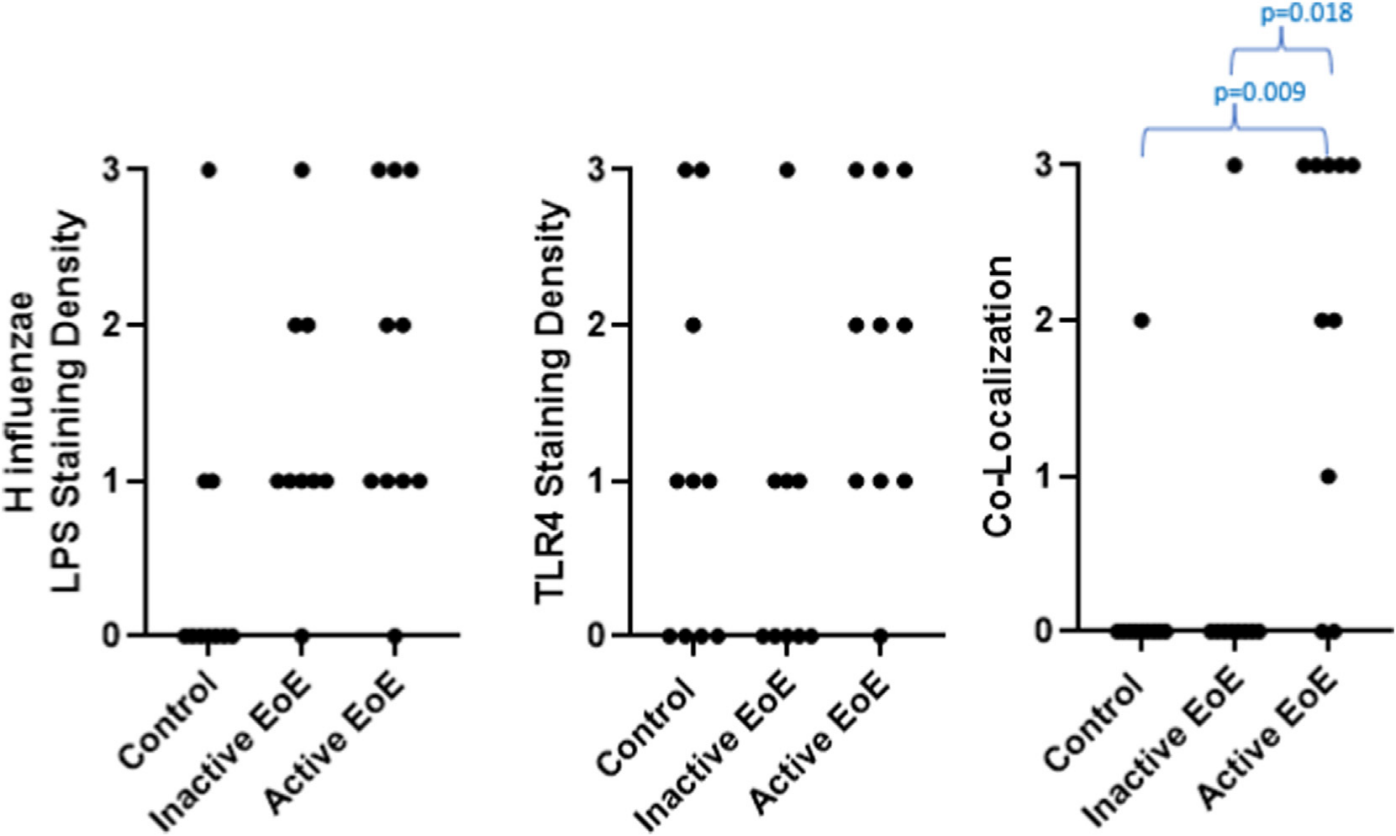
Graphic plots of LPS, TLR4, and colocalization of LPS and TLR4 in the controls, patients with inactive EoE, and patients with active EoE.

The mean age (range) of the patients with active EoE was 28 years (18-59 years), that of the patients with inactive was EoE 36 years (range 22-67 years), and that of the of control patients was 53 years (range 25-83 years). All of the patients were White. Of the 17 patients with EoE, 7 achieved remission with proton pump inhibitors (PPIs) (Table 1). Of the patients with active EoE, 2 were receiving diet exclusion therapy, 4 were being treated with topical steroids, and 4 were not receiving any therapy at the time of sample collection. Of the patients with inactive disease, 3 were being treated with diet exclusion therapy and 6 were being treated with PPIs at the time of sample collection. *H influenzae* LPS was detected by positive staining in 20 of the 29 patients (69.0%), including 9 of the 10 with active EoE (90.0%), 8 of the 9 with inactive EoE (89.9%), and 3 of the 10 control patients (30%) (Tables II and III and Fig 1, *A*). The pattern of staining demonstrated focal to diffuse cellular membrane staining. Whether this was cytoplasmic or membranous staining could not be determined. Staining intensity was greater in the samples from patients with active EoE than in the samples from the control patients, although the difference was not statistically significant (*P* = .063 [Tables II and III and Fig 2]).

**Table I:** Characteristics of patients with EoE and controls

Activity	Age	Sex/race	Atopy	Peak eos count (eos/hpf)	EREFS	PPI responder	Food responder	Steroid responder
Active **(**n = 10**)**	42	M/W	N	65	3	N	N	Y
	35	M/W	AR, DA, FA (egg)	15	3	N	Milk/wheat/soy/egg	—
	37	F/W	A, DA	>100	3	N	Y Legumes, wheat, tree nuts, peanut	—
	44	F/W	A	82	N/A	N	—	Y
	18	M/W	A, AR	80	N/A	Y	—	—
	53	F/W	FA (fish, milk, nuts)	40	3	N	Y	Y
	24	F/W	AR	54	N/A	N	Y	—
	59	F/W	AR s/p AIT	40	4	N	—	Y
	40	F/W	Asthma, AR, DA	>100	3	N	N	—
	42	F/W	AR	35	2	N	N	N
Inactive **(**n = 9**)**	22	F/W	AD, AR	0	N/A	Y	—	—
	54	M/W	N	0	1	Y	—	—
	67	F/W	AR.	12	0	Y	—	—
	60	M/W	N	10	0	Y	—	—
	30	M/W	FA, DA,	3	N/A	N	Y	—
	47	F/W	DA	0	N/A	Y	—	—
	51	F/W	DA, AR	2	N/A	N	Y	—
	24	M/W	AD, AR	1	N/A	Y	—	—
	57	F/W	DA, FA, A	0	2	N	Y	—
Control n=10	73	M/W	AR, A, DA	0				
	83	M/W	DA	0				
	81	F/W	N	0				
	25	F/W	N	0				
	65	F/W	DA	0				
	30	F/W	N.	0				
	43	F/W	DA	0				
	60	M	DA, AR	0				
	40	F/W	A	0				
	26	M/W	N	0				

Responder denotes EoE remission, regardless of therapy at the time of sample collection.

*A*, Asthma, *AIT*, allergen-specific immunotherapy; *AR*, allergic rhinitis; *DA*, drug allergy; *eos*, eosinophil; *EREFS*, Eosinophilic Esophagitis Endoscopic Reference Score; *F*, female; *FA*, food Allergy; *M*, male; *N*, no; *NA*, not available; *W*, White; *Y*, yes.

**Table II:** Summary data for LPR, TLR4, and colocalization in patients with active EoE, patients with inactive EoE, and controls

Patient group	Staining density			Max eos count (eos/hpf)
	LPS	TLR4	Colocalization	
With active EoE (n = 10)	1	1	1	65
	0	0	0	15
	1	2	2	>100
	2	2	2	82
	3	3	3	80
	2	2	3	40
	1	1	0	54
	3	3	3	40
	3	3	3	>100
	1	1	3	>35
Control (n = 10)	0	0	0	0
	1	0	0	0
	0	1	0	0
	3	3	0	0
	0	1	0	0
	0	0	0	0
	0	0	0	0
	1	1	2	0
	0	2	0	0
	0	3	0	0
With inactive EoE (n = 9)	2	0	0	0
	1	1	0	0
	1	0	0	11
	1	0	0	10
	0	0	0	3
	1	1	0	0
	3	3	3	2
	2	0	0	1
	1	1	0	0

**Table III:** Summary data for LPR, TLR4, and colocalization in patients with active EoE, patients with inactive EoE, and controls

Staining density	Control (n = 10)	With inactive EoE (n = 9)	With active EoE (n = 10)	*P* value
LPS, no. (%)				.037
0	7 (70.0%)	1 (11.1%)	1 (10.0%)	
1	2 (20.0%)	5 (55.6%)	4 (40.0%)	
2	0 (0.0%)	2 (22.2%)	2 (20.0%)	
3	1 (10.0%)	1 (11.1%)	3 (30.0%)	
TLR 4, no. (%)				.092
0	4 (40.0%)	5 (55.6%)	1 (10.0%)	
1	3 (30.0%)	3 (33.3%)	3 (30.0%)	
2	1 (10.0%)	0 (0.0%)	3 (30.0%)	
3	2 (20.0%)	1 (11.1%)	3 (30.0%)	
Colocalization, no. (%)				.002
0	9 (90.0%)	8 (88.9%)	2 (20.0%)	
1	0 (0.0%)	0 (0.0%)	1 (10.0%)	
2	1 (10.0%)	0 (0.0%)	2 (20.0%)	
3	0 (0.0%)	1 (11.1%)	5 (50.0%)

TLR4 was detected by positive staining in 19 of the 29 patients (65.5%), including 9 of the 10 with active EoE (90.0%), 4 of the 9 with inactive EoE (44.4%), and 6 of the 10 control patients (60.0%) (Tables II and III and Fig 1, *B*). The pattern of distribution of TLR4 appeared similar to that of LPS, ranging from focal to diffuse. The staining was assumed to be membranous, as the epithelial cell membrane is the location of the TLR4 receptor. The pattern appeared similar to that of LPS. There was greater TLR4 staining density in patients with active EoE than in those with inactive EoE, although the difference was not statistically significant (*P* = .096 [Tables II and III and Fig 2]).

Colocalization of *H influenzae* LPS and TLR4 staining was assessed because, as previously discussed, LPS has been demonstrated to be a primary activator of TLR4. Colocalization was positive in 10 of 29 patients overall (34.5%), including 8 of the 10 with active EoE (80.0%), 1 of the 9 with inactive EoE (11.1%), and 1 of the 10 control patients (10.0%) (Tables II and III and Figs 1 and 2). There was a statistically significant difference between colocalization of *H influenzae* LPS and TLR4 staining density in the patients with active EoE and that in the control patients and patients with inactive EoE (*P* = .009 and *P* = .018, respectively).

Increased expression of TLRs and an abnormal esophageal microbiome have both been demonstrated in EoE, although the relationship between the 2 is unclear. In this study, it was demonstrated that there is a significant increase in both LPS and TLR4, with colocalization in patients with active EoE. These data provide a potential link between the esophageal microbiome and activation of the innate immune system in the pathogenesis of EoE.

LPS is an important mediator of inflammation. For example, in Crohn disease, lumen dysbiosis LPS colocalizes with TLR4 to perpetuate chronic inflammation.^11^ Several questions arise in applying this pathway to the esophagus. The first is the question of how LPS is produced in the esophagus. *H influenzae* is present in normal esophageal flora; its level is increased in patients with eosinophilic esophagitis, and it is an important source of LPS^2^^,^^4^^,^^14^ Furthermore, an increased abundance of *H influenzae* in the esophageal microbiome corresponds to increased disease activity.^2^ This model of an increased *H influenzae* abundance contributing to a T_H_2 pathway–mediated disease has been demonstrated in patients with asthma^15^ and chronic rhinosinusitis.^16^

Another key question is the mechanism by which can LPS penetrate the relatively impermeable esophageal epithelium. When degraded, LPS has a molecular mass of approximately 50 to 100 kDa.^17^ The latter is a weight to similar that of gliadin, which also is also characterized by selective penetration of esophageal epithelium related to EoE activity, in which the epithelium is more permeable. This may be related to dilation of intercellular spaces. We have previously demonstrated that the degree of intercellular space dilation corresponds to selective entry of food antigens based on size.^18^^,^^19^ One might also postulate that increased access of the microbiome to the esophageal mucosa is facilitated by the absence of a thick mucus layer versus in the intestine.

Third, there is the question of the cell in which binding of LPS to TLR4 occurs. Several candidate cells have been studied as an antigen-presenting cell in EoE; they include dendritic cells, eosinophils, T_H_2 cells, and epithelial cells. At this point, which cell(s) perform this function is unclear. Of additional note is the finding of TLR4 binding in some normal controls, implying that is it not so much the presence as the colocalization and potential binding to LPS that is functionally important.

This study has several limitations. The first is a small sample size and use of nonpaired samples to compare patients with active disease and with patients in remission. On the other hand, this model has been successfully used to demonstrate significant differences in previous studies, as the activity of EoE is standardized by clear histologic criteria.^20^ There is also the concern that EoE therapies could affect the esophageal microbiome. Nevertheless, previous studies have not demonstrated an effect of diet, PPI, or steroid therapy on esophageal microbiota,^5^^,^^14^ nor have any of studies found that any of these factors lead to different histology or function of the esophageal epithelium with healing. Conversely, emerging data indicate that PPIs are effective in EoE through proton pump–independent pathways such as cytokine inhibition,^21^ restoration of normal esophageal permeability,^22^ and broad transcriptional changes in the esophageal epithelium.^23^ These mechanisms may help to restore esophageal permeability similar to the effect of steroids in EoE. This raises the question of whether the therapy in patients with EoE potentially blunted our results by sustaining a partial response. This is unlikely, as 9 of the 10 patients with active EoE had an eosinophil count higher than 35 per hpf and 4 of the 10 had had an eosinophil count higher than 80, indicating at least moderately active disease. Another limitation is that our measurement of staining intensity is not as quantitative as PCR assay of the tissue might be. With this technique, however, one cannot be sure of the uniformity of the biopsy sample with respect to measurement of only the mucosal content of the marker of interest. Neither does it give us the vital information on colocalization. This study also used distal esophageal biopsy samples exclusively. On the other hand, histologically and functionally, no differences between different esophageal locations consistent with the pan-esophageal dysfunction seen in EoE have been demonstrated. Finally, our control group was older than the group of patients with EoE, arousing concerns that this could affect our study's results, particularly results related to the microbiota population. Conversely, the structure of the gut microbiota is well known to be relatively stable throughout adulthood. The ageing process starts to affect the gut microbiota after individuals reach the age of 65 years.^24^

Nevertheless, whether and in what capacity the presence of LPS promotes T_H_2 pathway activation in response to food antigen exposure is unclear. As the presence of specific food antigens such as gluten or milk protein is required to sustain the inflammatory response in EoE in most patients, LPS may act as a cofactor derived from microbially divergent microbiome. The presence of such a cofactor or priming mechanism would fit with previous data demonstrating that patients with EoE and normal controls may have food antigen present in the mucosa without active inflammation and/or eosinophilia.^18^ It is also possible that the presence of LPS in EoE is facilitated by underlying damage to the epithelium by the microbiota,^25^ but this would not explain the colocalization with TLR4 seen here in patients with active EoE. Finally, the presence of LPS may potentially promote the T_H_1 pathway via TLR4, as seen with inhaled antigens in asthma,^26^ which may potentially antagonize the T_H_2 pathway in EoE. We believe that this is unlikely to be the dominant pathway of LPS activation of inflammation.

In conclusion, these data demonstrate active colocalization of TLR and LPS in patients with EoE. Furthermore, the presence of both TLR and LPS and the degree of colocalization appear dependent on EoE activity. This gives further support to a role of the esophageal microbiome in influencing activity in EoE. Further investigation is needed to establish whether this colocalization represents binding with more direct activation of the T_H_2 pathway and whether this finding is initiated by the presence of increased levels of *H influenzae* in the esophageal microbiome.

## Disclosure statement

Disclosure of potential conflict of interest: The authors declare that they have no relevant conflicts of interest. D. A. Katzka reports consulting for Takeda, Regeneron, and Celgene. The rest of the authors declare that they have no relevant conflicts of interest.

**Clinical implications**

This study adds support to the link between esophageal *H influenzae* LPS and TLR4, a key messenger in the T_H_2 pathway in EoE. These findings provide a potential future microbial target for therapy in EoE.
