# Supplementary figures and images for: H influenzae LPS colocalization with Toll-like receptor 4 in eosinophilic esophagitis
Source: J Allergy Clin Immunol Glob. 2023 Jul 20;2(4):100151. doi: 10.1016/j.jacig.2023.100151 (PMC10679775; doi:10.1016/j.jacig.2023.100151)

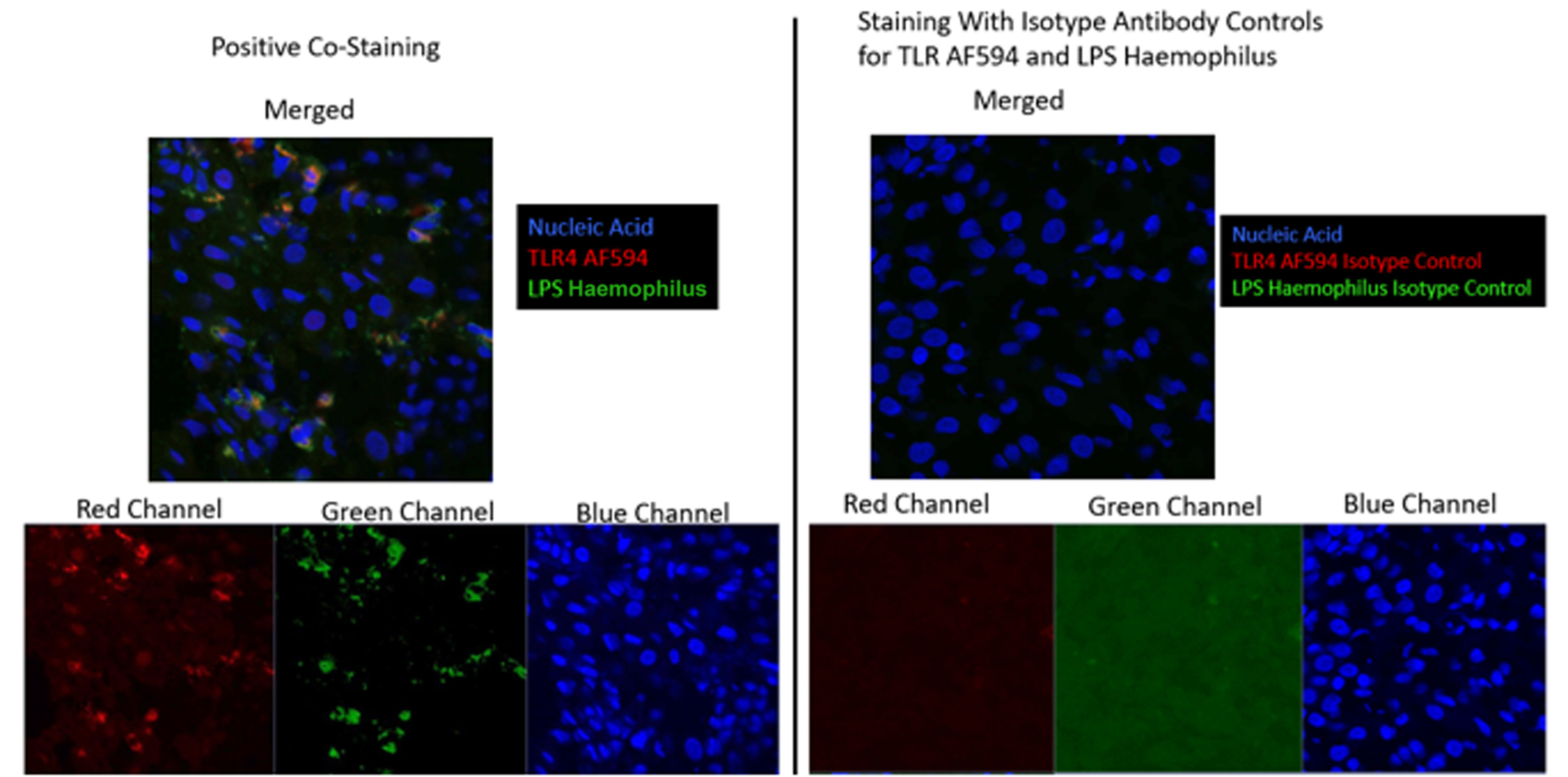

Supplement: Supplementary Figure 1 [file figs1.jpg]
