# Supplementary material for: H influenzae LPS colocalization with Toll-like receptor 4 in eosinophilic esophagitis
Source: J Allergy Clin Immunol Glob. 2023 Jul 20;2(4):100151. doi: 10.1016/j.jacig.2023.100151 (PMC10679775; doi:10.1016/j.jacig.2023.100151)
Supplement: Supplementary Material [file mmc1.docx]

**Supplementary Methods:**

Cryosectioned slides were allowed to come to room temperature, and then washed twice (5min incubation time each) in 1x PBS. Blocking Buffer of 2% BSA/PBS was added for 1 hour. Slides were washed 2x as described above (ADA). FC receptor blocking agent (Innovex Biosciences)  was added for  30 min. 2x washes in PBS (ADA). Purified anti LPS H. influenza (NovusBio) was added at a 1/100 dilution and incubated for 1hour. 3x washes in PBS (ADA). Fitc conjugated anti mouse IgG (Jackson ImmunoResearch Laboratories) was added at a 1/200 dilution and incubated for 1 hour. 3x washes in 1x PBS. (ADA) An additional blocking step consisting of purified mouse IgG (Jackson ImmunoResearch Laboratories) (150ug/slide)  was done for 1hour. 3x washes in 1x PBS. (ADA) Alexa Fluor 594 (AF594) conjugated anti TLR4 (1/100 dilution) was added for 1 hour. 3x washes in 1x PBS (ADA). Dapi incubation was done for 20 minutes. 3x washes in 1x PBS (ADA). Residual buffer was aspirated and mountant buffer (Invitrogen SlowFade Gold Antifade) was added. Coverslips were added and sealed with nail polish.

**Supplementary Figure Legend**

Figure S1: Isotype control staining for a biopsy with positive co-staining
